# Supplementary material for: Complexome profiling of the Chlamydomonas psb28 mutant reveals TEF5 as an early PSII assembly factor
Source: Plant Cell. 2025 Mar 17;37(6):koaf055. doi: 10.1093/plcell/koaf055 (PMC12164590; doi:10.1093/plcell/koaf055)
Supplement: koaf055_Supplementary_Data [file koaf055_supplementary_data.zip › psb28_interactive.zip!/plots/intensity/qualityPlots/peptides/FBP2_Cre12.g510650.t1.2.html]

### Cre12.g510650.t1.2

Trivial name: FBP2  
SeqCoverage [median]: 8.70  
Mass: 44.44 kDa  
  
 

| Sequence | MissedCleavage | Mass [Da] | wtIntensity | muIntensity | Score |
| --- | --- | --- | --- | --- | --- |
| TLLYGGIYGYPGDAK | 0 | 1586.80 | 0.00 | 465.63 | 76.65 |
| YIGSLVGDFHR | 0 | 1262.64 | 0.00 | 1015.80 | 56.36 |
|  |  |  | 0.0 | 1481.4 |  |
